# Analysis of *KLF *transcription factor family gene variants in type 2 diabetes

**DOI:** 10.1186/1471-2350-8-53

**Published:** 2007-08-09

**Authors:** Ruth Gutiérrez-Aguilar, Yamina Benmezroua, Emmanuel Vaillant, Beverley Balkau, Michel Marre, Guillaume Charpentier, Rob Sladek, Philippe Froguel, Bernadette Neve

**Affiliations:** 1 UMR8090, CNRS, Institute of Biologie/Institute Pasteur de Lille, Lille, France; 2 Université de Lille 2, Lille, France; 3 INSERM, U780-IFR69, Villejuif, France; 4 U780-IFR69, Université Paris-Sud, Villejuif, France; 5 U695, INSERM, Xavier Bichat Faculty of Medicine, Université Paris 7, Paris, France; 6 Hospital Bichat – Claude Bernard, Paris, France; 7 Departement of Diabetes, Sud Francilien Hospital, Corbeil-Essonnes, France; 8 Human Genetics and Medicine, Faculty of Medicine, McGill University, Montreal, Canada; 9 Genome, Quebec Innovation Centre, Montreal, Canada; 10 Genomic Medicine, Hammersmith Hospital, Imperial College London, UK; 11 Genomic Medicine, Hammersmith Hospital, Imperial College, Du Cane Road, London W12 0NN, UK

## Abstract

**Background:**

The Krüppel-like factor (*KLF*) family consists of transcription factors that can activate or repress different genes implicated in processes such as differentiation, development, and cell cycle progression. Moreover, several of these proteins have been implicated in glucose homeostasis, making them candidate genes for involvement in type 2 diabetes (T2D).

**Methods:**

Variants of nine *KLF *genes were genotyped in T2D cases and controls and analysed in a two-stage study. The first case-control set included 365 T2D patients with a strong family history of T2D and 363 normoglycemic individuals and the second set, 750 T2D patients and 741 normoglycemic individuals, all of French origin. The SNPs of six *KLF *genes were genotyped by Taqman^® ^SNP Genotyping Assays. The other three *KLF *genes (KLF2, -15 and -16) were screened and the identified frequent variants of these genes were analysed in the case-control studies.

**Results:**

Three of the 28 SNPs showed a trend to be associated with T2D in our first case-control set (P < 0.10). These SNPs, located in the *KLF2, KLF4 *and *KLF5 *gene were then analysed in our second replication set, but analysis of this set and the combined analysis of the three variants in all 2,219 individuals did not show an association with T2D in this French population. As the *KLF2*, -15 and -16 variants were representative for the genetic variability in these genes, we conclude they do not contribute to genetic susceptibility for T2D.

**Conclusion:**

It is unlikely that variants in different members of the *KLF *gene family play a major role in T2D in the French population.

## Background

The Krüppel-like factor (*KLF*) proteins belong to a family of transcription factors that bind to GC-GT rich sites and CACC boxes of a large range of gene promoters [1-4]. Binding of these factors is mediated by a highly conserved DNA-binding motif of C2H2 zinc fingers localised at their C-terminus [5], which suggests that these proteins could have a redundant activity. However, accumulated evidence shows that each *KLF *has its own function and regulation [6,7]. *KLF *transcription factors act as transcriptional activators or repressors depending on the specificity of promoters to which they bind and the cellular context. Moreover, they interact with various co-activators or co-repressors, regulating via different mechanisms, the transcription of their target genes [4].

We recently identified both rare and frequent genetic variants in KLF11 co-segregating with early onset familial diabetes or associated with late-onset T2D [8]. These results prompted us to assess the putative genetic contribution to T2D risk of additional *KLF *family members, especially those presumably involved in energy and glucose homeostasis, through their expression in the pancreas, adipose tissue, liver and muscle [9-11]. Indeed, KLF2, *KLF5*, and *KLF6 *have been suggested to be involved in adipocyte differentiation [12-14]; KLF2 and *KLF15 *may contribute to adipogenesis transcriptional regulation via PPARγ signalling [15,16]. In particular, this implication in the differentiation process of adipocytes, suggests they may play an important role in insulin resistance, a status prone to T2D development.

Thus, the possible implication of the *KLF *family in glucose homeostasis and adipogenesis makes its members relevant gene candidates for T2D. Therefore, we assessed if frequent genetic variants in several members of the *KLF *family are implicated in genetic susceptibility to T2D development in a French population. To assess the putative contribution of genetic variability of KLFs gene family members to type 2 diabetes (T2D) we performed a two-stage association study.

## Methods

### Subjects

Normoglycemic and type 2 diabetic subjects were defined according to World Health Organization criteria. The characteristics of the first case-control study, summarized in table 1, included 365 French T2D unrelated patients with at least one affected first-degree relative (D1) and 363 non diabetic, unrelated spouses from T2D families (C1) recruited by the "Centre National de la Recherche Scientifique"-Institute Pasteur Unit in Lille. The second sample set (table 1) included 750 T2D patients (D2) recruited at the Endocrinology-Diabetology Department of the Corbeil-Essonne Hospital, and 741 control individuals (C2) from the D.E.S.I.R. study (Données Epidemiologiques sur le Syndrome d'Insulino-Resistance [17]).

**Table 1:** General characteristics of the French populations studied

	**Case-control Set 1**		**Case-Control Set 2**	
D, type 2 diabetic patients; C, control individuals	**(D1)**	**(C1)**	**(D2)**	**(C2)**
Numbers studied	365	363	750	741
Sex (men/women)	196/169	136/227	455/295	294/447
Age at examination (years, mean ± SD)	-	59 ± 13	-	53 ± 6
Age at diagnosis of diabetes (years, mean ± SD)	45 ± 11	-	48 ± 10	-
BMI (kg/m^2^, mean ± SD)	26.8 ± 3.8	25.4 ± 4.7	33.0 ± 6.4	23.0 ± 1.8

### SNP screening and genotyping

Single nucleotide polymorphisms (SNPs) in putative linkage disequilibrium blocks of the *KLF3*, *KLF4*, *KLF5, KLF6, KLF12*, and *KLF13 *genes were chosen from available Taqman^® ^assays (SNP browser 3.0) and genotyped by Taqman^® ^SNP Genotyping Assay (Applied Biosystems, Foster City, CA, U.S.A.). For *KLF*15 and *KLF*16 genes, no Taqman^® ^probes were available from the SNP Browser 3.0. We screened the *KLF*2, *KLF*15 and *KLF*16 genes for variants by direct sequencing of the promoter region (up to 1 kb), the 5' and 3' UTRs, exons and flanking intron sequences of the genes in DNA samples from 32 unrelated individuals using the ABI Prism 377 DNA sequencer. The identified SNPs were genotyped by Light Cycler/Lightyper technology (Roche Diagnostic, Basel, Switzerland). SNPs were genotyped in the first case-control set and those with a minor allele frequency over 10% (MAF>10%) that showed a trend to be associated with T2D (P < 0.10) were further studied in the second case-control set. Duplicate measurements were included to validate the genotype scores with 100% of concordance.

### Statistical analysis

SNPs of several *KLF *family members were genotyped in the first case-control set of 728 individuals. Comparison of allelic frequencies between cases and controls used a χ2 test with the Pearson P-value under an allelic model (FINETTI program [18]). The Odds Ratios (ORs) with 95% confidence intervals (CIs) for allelic effects were calculated with the same program. In the first set, D1/C1, the statistical power, calculated with the PAWE program [19] to detect an OR of 1.4, was 68% to 84% (α = 0.05, minor allele frequency (MAF) = 0.15 to 0.20). To increase the power, we selected SNPs with a significance level of P < 0.10 for further analysis (power = 78% to 90%). The power to detect an OR of 1.29, previously reported for KLF11 [8], is 57% to 65% in this set (MAF = 0.15 to 0.20). We genotyped the selected SNPs in the set D2/C2 of 1,491 individuals. The power of the overall study (D/C) with 2,219 individuals was 99% for an OR = 1.4 (α = 0.05, MAF = 0.15) and over 84% for the observed ORs (0.75–1.27) of the selected SNPs in first set D1/C1.

## Results

In the first case-control set, we genotyped 28 frequent variants of 9 different members of the KLF gene family with a minor allele frequency (MAF) above 0.10 (table 2). All SNPs were in Hardy-Weinberg equilibrium according to the FINETTI program (data not shown). In table 2, we present the NCBI reference number (dbSNP ID) and details for each SNP genotyped, as well as the MAF and the P-value under an allelic model. From this first study set (D1/C1), three SNPs showed an association with T2D; *KLF2 *rs12459387 (P = 0.09, MAF = 0.12, OR = 0.75 with 95% CI = 0.53–1.05), *KLF4 *rs10759240 (P = 0.08, MAF = 0.52, OR = 0.83 with 95% CI = 0.67–1.02) and *KLF*5 rs11841945 (P = 0.04, MAF = 0.40, OR = 1.27 with 95% CI = 1.02–1.59).

**Table 2:** Genotypic distribution of KLF SNPs in type 2 diabetic and non-diabetic individuals

**dbSNP ID**	**N/M**	**MAF D1/C1**	**P-value**	**MAF D2/C2**	**P-value**	**MAF D/C**	**P-value**
**KLF2, Chr. 19q13**		0.31 (167, 142, 34)	0.69				
Prom -789	C/T	0.32 (148, 143, 30)					
rs10424657	G/A	0.30 (175, 146, 34)	0.61				
		0.31 (162, 155, 32)					
rs12459387	C/T	0.09 (292, 58, 4)	0.09	0.10 (464, 106, 6)	0.87	0.10 (756, 164, 10)	0.40
		0.12 (260, 78, 2)		0.10 (543, 123, 6)		0.11 (803, 201, 8)	
rs7248864	C/T	0.09 (295, 56, 5)	0.13				
		0.12 (270, 78, 2)					
rs3745318	C/T	0.21 (211, 109, 16)	0.67				
		0.20 (214, 114, 11)					
rs12462380	A/G	0.41 (119, 178, 56)	0.24				
		0.44 (102, 170, 63)					
rs7258799	A/T	0.13 (277, 81, 6)	0.15				
		0.16 (219, 74, 10)					
rs15336	C/A	0.19 (235, 111, 12)	0.21				
		0.16 (235, 94, 8)					
rs11086029	A/T	0.22 (209, 121, 14)	0.63				
		0.21 (217, 117, 13)					

**KLF3, Chr. 4q14**		0.44 (110, 180, 70)	0.89				
rs2045767	G/T	0.45 (105, 182, 68)					
rs9522	C/T	0.45 (110, 171, 75)	0.62				
		0.46 (97, 178, 72)					

**KLF4, Chr. 9q31**		0.47 (97, 174, 79)	0.08	0.49 (144, 297, 133)	0.68	0.48 (241, 471, 212)	0.08
rs10759240	C/A	0.52 (80, 168, 95)		0.51 (176, 361, 187)		0.51 (256, 529, 282)	

**KLF5, Chr. 13q21**		0.16 (248, 97, 6)	0.67				
rs12428414	C/T	0.16 (237, 88, 11)					
rs4885065	A/G	0.17 (245, 94, 11)	0.83				
		0.16 (247, 98, 8)					
rs9564944	A/G	0.12 (263, 69, 5)	0.90				
		0.12 (269, 62, 8)					
rs11841945	C/G	0.46 (94, 173, 65)	0.04	0.42 (200, 271, 110)	0.20	0.43 (294, 444, 175)	0.91
		0.40 (110, 145, 48)		0.45 (227, 349, 151)		0.43 (337, 494, 199)	

**KLF6, Chr. 10p15**		0.11 (290, 68, 5)	0.63				
rs7005	C/T	0.12 (281, 66, 8)					

**KLF12, Chr. 13q21**		0.36 (148, 164, 47)	0.36				
rs1927010	A/T	0.32 (154, 162, 38)					
rs4885151	G/T	0.31 (165, 166, 31)	0.80				
		0.32 (169, 144, 42)					
rs7993625	C/T	0.24 (205, 128, 21)	0.60				
		0.23 (199, 133, 12)					
rs6650443	A/G	0.19 (245, 99, 19)	0.66				
		0.20 (224, 107, 15)					
rs9600167	C/G	0.35 (154, 158, 46)	0.49				
		0.23 (160, 141, 44)					

**KLF13, Chr. 15q12**		0.44 (120, 169, 74)	0.71				
rs11855557	A/G	0.45 (102, 189, 64)					
rs12592176	G/A	0.50 (84, 186, 83)	0.49				
		0.48 (98, 172, 84)					
rs4779516	T/C	0.44 (115, 175, 70)	0.36				
		0.46 (96, 187, 69)					

**KLF15, Chr. 3q13**		0.12 (268, 74, 4)	0.35				
3'UTR+244del(CACGAAGG)		0.10 (285, 58, 7)					

**KLF16, Chr. 19q13**		0.47 (100, 162, 79)	0.11				
rs11670146	A/G	0.47 (101, 144, 56)					
rs3746045	T/C	0.24 (208, 120, 24)	0.23				
		0.21 (214, 119, 14)					

D, type 2 diabetic patients; C, non-diabetic individuals; MAF, minor allele frequency; (number of subjects with wild type homozygote; heterozygote; mutant homozygote alleles); P-value for differences between allele frequencies under an allelic model.

In the second case-control set, we genotyped *KLF2 *rs12459387, *KLF4 *rs10759240 and *KLF5 *rs11841945, but the observed difference in MAF between cases and controls in D1/C1 was not confirmed (Table 2). In fact, the trend for allelic association with T2D observed in the first set was not replicated for these SNPs in the second set (D2/C2), nor were they significant after the combined analysis of both groups (D/C). As D2 had a higher mean body mass index (BMI) compared to D1, we also analyzed the data of individuals with BMI < 27 kg/m^2 ^but still no significant difference was observed. In addition, we did not observe associations between these three SNPs and quantitative traits such as fasting insulin, fasting glucose, OGTT.

Since our study is probably limited by the fact that it does not cover all the genetic variability of the KLF genes, we analysed additional SNPs included in our recently published genome wide association study [20]. The SNPs located in regions spanning the KLF genes, and analyzed in this genome wide association study (148) showed no significant association with T2D in this French population (data not shown).

## Discussion

We have analysed SNPs within nine KLF genes and we were not able to show a genetic T2D association in our population. A similar study in a Japanese population also showed no evidence for an association with other variants of the same *KLF *genes analysed in our study (*KLF2*, -3, -5, -12, -13 and *KLF16 *[21]). However, it is worth noting that some *KLF *genes are not covered extensively with HapMap genotyped SNPs. In fact, this was the case for *KLF*15 and *KLF*16 gene regions, where at the start of this study no Taqman^® ^assays were available. Therefore, we decided to screen these genes for common SNPs. Since we analysed in our study all frequent SNPs without any positive results, we conclude that the *KLF*15 and *KLF*16 genes are not likely to be associated with T2D in a French population.

A limitation of our study is the two-staged design, through which we may have missed a weak but true association with T2D. However, the use of T2D patients with affected first degree relatives and controls sharing the same environment as the cases in set 1, probably increased our power to detect an association with T2D. A second limitation of this study is the fact that it does not cover all the genetic variability of the *KLF *genes. However, our subsequent genome wide association analysis with 1,363 individuals also did not show significant T2D associations for variants of these genes, making it unlikely the nine KLFs play a major role in genetic T2D susceptibility.

We hypothesised that *KLF *genes could be associated with genetic susceptibility for T2D, based on their role in adipogenesis. In particularly, *KLF2 *is implicated in the regulation of PPARγ [15], which is a target gene implicated in the genetic susceptibility for T2D. Therefore, we studied this gene more extensively and genotyped 9 SNPs. Also for *KLF*2 variants, we did not observe an association with T2D in this French population. In addition, a previous study of *KLF2 *variants and body weight control in another French population did not observe an association with adipose tissue-related traits [22]. From these results, it seems unlikely that the genetic variability of the KLF2 gene plays a mayor role in susceptibility to T2D or obesity.

## Conclusion

In our study of variants from nine different members of the *KLF *gene family, we observed no association with T2D in our large French population. Moreover, the genotyped SNPs covered the overall genetic variability of the *KLF2*, -*1*5 and -*16 *genes so we may conclude that these genes do not contribute to the genetic susceptibility of T2D.

## Competing interests

The author(s) declare that they have no competing interests.

## Authors' contributions

RGA wrote the paper. RGA and BN performed the statistical analyses. YB and EV genotyped the variants of this study. RS provided data and statistical analyses of the genome wide association study. BB, MM, GC and PF established the diverse DNA collection used. PF and BN directed the study and contributed to the redaction of the paper.

## Pre-publication history

The pre-publication history for this paper can be accessed here:


